# SIMLive! Scaling‐up simulation for contextually rich clinical reasoning

**DOI:** 10.1111/medu.15649

**Published:** 2025-03-04

**Authors:** Leah Williams

## 1 WHAT PROBLEMS WERE ADDRESSED?

Integrating clinical reasoning (CR) training into medical school curricula is becoming increasingly important. As CR acumen is developed iteratively, and often experientially, early introduction of such training gives students greater opportunity to refine their skills. By facilitating explicit CR, high‐fidelity simulation (HFS) is an ideal pedagogical tool to address this need. However, traditional sessions are resource‐intensive, requiring repeated small‐group sessions and significant educator time. At the University of Sunderland, HFS was previously limited to clinical years (3–5), leaving first‐ and second‐year students with little exposure to this method of teaching despite its demonstrable benefits. A scalable, authentic and resource‐efficient solution was needed to introduce HFS into pre‐clinical teaching, while fostering early CR skills and maintaining student engagement.

## 2 WHAT WAS TRIED?

SIMLive! was developed to solve this problem by integrating HFS (using SIM‐Man 3G) with a real‐time, single‐best‐answer quiz that allows students to apply pre‐clinical knowledge in a stimulating way. This novel format was delivered to approximately 100 second‐year students in a 2.5‐hour workshop, focussing on the ABCDE assessment^1^ and treatment of a patient with asthma. The session, case, and quiz were designed and delivered by LW, a practicing clinician with CR research experience. The simulation was positioned at the centre of an auditorium, with patient observation screens dispersed around the room. Auscultation sounds were played aloud to further immerse students in the experience. Periodically, the simulation paused, and students answered questions anonymously on their devices, with feedback and explanations provided immediately afterwards. Questions ranged from basic recall to more advanced application of pharmacology, physiology and interpretation skills. Pre‐simulation group discussions were conducted to activate prior knowledge, and post‐simulation teaching helped to consolidate learning. To benefit the wider curriculum, SIMLive! is now being successfully adapted to sessions across teaching units by interchanging relevant cases and quiz questions, creating a spiral curriculum approach.

## 3 WHAT LESSONS WERE LEARNED?

Feedback was collected using Likert scales and free‐text boxes. Most learners valued the approaches used, and 99% ‘agreed’/‘strongly agreed’ that their understanding of the topic was improved by the session. One key lesson taken from this feedback was that integrating simulation with a quiz enhances student engagement. Free from the characteristic ‘performance anxiety’ observed in small‐group simulation, students viewed this anonymised method of simulation‐based formative assessment as an inviting learning opportunity. Furthermore, by introducing critical thinking *alongside* the ABCDE framework, SIMLive! presents the added benefit of priming students for CR *during* simulation, a distinct benefit over traditional methods of HFS.

These findings suggest that SIMLive! is a useful approach to scaffolding both clinical confidence and CR in medical students. Furthermore, the efficiency gains from SIMLive! were substantial, reducing a multi‐day simulation commitment into a streamlined event delivering the same learning outcomes. Suggested areas for improvement included longer quiz response times (to promote thorough reasoning) and clearer views of the simulation for some students. Students requested an additional break post‐simulation to allow for reflection on the scenario before consolidating learning. These insights were invaluable for subsequent refinements.

Overall, SIMLive! proved to be a successful model for interfacing the conventionally separate phases of pre‐clinical and clinical teaching.

## AUTHOR CONTRIBUTIONS

**Leah Williams:** Conceptualization; project administration; writing – original draft; writing – review and editing.

## CONFLICT OF INTEREST STATEMENT

None.

## ETHICS STATEMENT

Ethical approval was not required for this submission.

## Data Availability

The data that support the findings of this study are available from the corresponding author upon reasonable request.

## References

[1] Resuscitation Council UK . The ABCDE Approach [Internet]. Resuscitation Council UK. 2015 [cited 2024 Sep 12]. https://www.resus.org.uk/library/abcde-approach

